# Offshore Wind Energy and Marine Biodiversity in the North Sea: Life Cycle Impact Assessment for Benthic Communities

**DOI:** 10.1021/acs.est.2c07797

**Published:** 2023-04-14

**Authors:** Chen Li, Joop W. P. Coolen, Laura Scherer, José M. Mogollón, Ulrike Braeckman, Jan Vanaverbeke, Arnold Tukker, Bernhard Steubing

**Affiliations:** † Institute of Environmental Sciences (CML), Leiden University, P.O. Box 9518, 2300 RA Leiden, The Netherlands; ‡ Wageningen Marine Research, P.O. Box 57, 1780 AB Den Helder, The Netherlands; § Aquatic Ecology and Water Quality Management Group, Wageningen University, Droevendaalsesteeg 3a, 6708 PD Wageningen, The Netherlands; ∥ Marine Biology Research Group (MARBIOL), Ghent University, Krijgslaan 281, 9000 Ghent, Belgium; ⊥ Operational Directorate Natural Environment, Marine Ecology and Management, Royal Belgian Institute for Natural Science, Vautierstraat 29, 1000 Brussels, Belgium; # Netherlands Organization for Applied Scientific Research, P.O. Box 96800, 2509 JE Den Haag, The Netherlands

**Keywords:** offshore wind farms, marine ecosystems, characterization factors, species richness, species abundance, seabed occupation, artificial reef, trawling avoidance

## Abstract

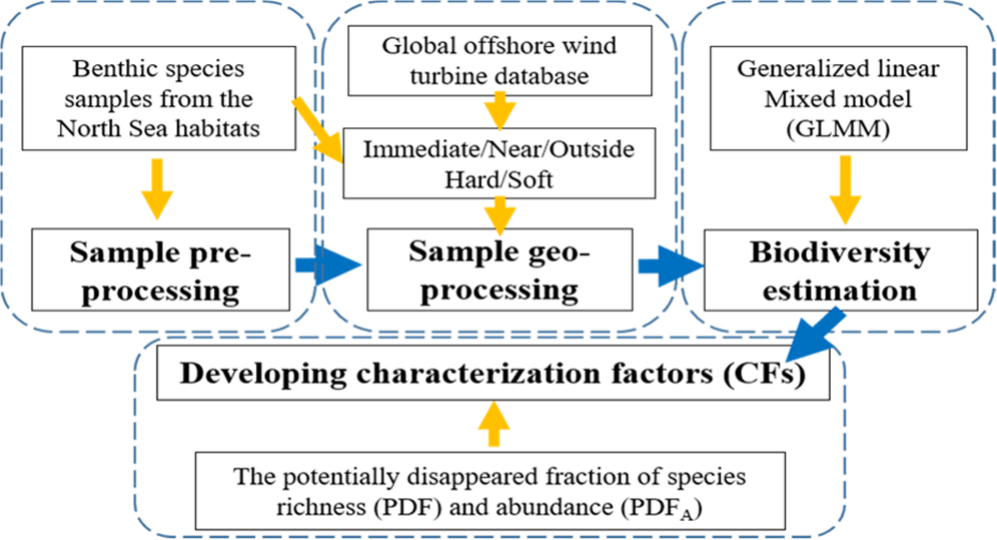

Large-scale offshore wind energy developments represent a major player in the energy transition but are likely to have (negative or positive) impacts on marine biodiversity. Wind turbine foundations and sour protection often replace soft sediment with hard substrates, creating artificial reefs for sessile dwellers. Offshore wind farm (OWF) furthermore leads to a decrease in (and even a cessation of) bottom trawling, as this activity is prohibited in many OWFs. The long-term cumulative impacts of these changes on marine biodiversity remain largely unknown. This study integrates such impacts into characterization factors for life cycle assessment based on the North Sea and illustrates its application. Our results suggest that there are no net adverse impacts during OWF operation on benthic communities inhabiting the original sand bottom within OWFs. Artificial reefs could lead to a doubling of species richness and a two-order-of-magnitude increase of species abundance. Seabed occupation will also incur in minor biodiversity losses in the soft sediment. Our results were not conclusive concerning the trawling avoidance benefits. The developed characterization factors quantifying biodiversity-related impacts from OWF operation provide a stepping stone toward a better representation of biodiversity in life cycle assessment.

## Abstract 2

This study develops a marine biodiversity impact assessment method for life cycle assessment and shows no net adverse impacts on benthic biodiversity during offshore wind energy operations.

## 1 Introduction

The North Sea holds approximately two-thirds (∼19 GW) of the globally installed offshore wind energy (OWE) capacity.^1^ The OWE industry is expected to progressively expand in the North Sea by 2050, with a target of 300 GW set by the European Commission.^2^ Such large-scale development would be a pillar for the energy transition and greenhouse gas emission mitigation but could have a substantial impact on marine biodiversity. Offshore wind turbines can cause collisions and a change in the migratory paths of seabirds and bats.^3^ Noise and vibration during offshore wind farm (OWF) installation and operation may affect fish and marine mammal communication and navigation.^4^ Electromagnetic fields generated by submarine cables may disturb foraging, orientation, and migration of fish species.^5−7^ Contrastingly, OWE infrastructure can also attract species for food or refuge (e.g., larger gulls,^8^ demersal and bentho-pelagic fish^9^). Since foundations occupy the seabed, they could have impacts on benthos.^10^ Foundations and scour protections are quickly colonized by hard substrate benthic species.^10,11^ Such artificial reefs can provide habitat and food for benthic communities. Further, bottom trawling in OWFs is typically not permitted, and the OWE development will therefore lead to bottom trawling free zones with effects that are potentially similar to fishery restrictions or conservation areas.^10^ These effects may have significant impacts on local ecosystems, yet the long-term cumulative impacts of large-scale projected OWFs on marine biodiversity remain largely unknown. In order to avoid shifting the burdens from greenhouse gas emission mitigation to marine biodiversity, there is a need to understand the local and cumulative impacts of OWE development on marine biodiversity.

Declines in marine biodiversity driven by human activities have been documented on a global scale.^12,13^ The environmental impact assessment framework has been used to discuss regulatory needs to prevent marine biodiversity loss from OWFs.^7,14,15^ Other studies have theoretically analyzed how OWE-relevant impacts can be assessed, including habitat change,^5,9,16−19^ electromagnetic fields,^5,9,16−18,20^ noise,^5,9,16−18,20^ artificial reefs,^9,10,18^ and fishery avoidance.^9,17,18^ However, quantitative assessments (and monitoring) of the impact of OWE development on marine life have been limited to only a few areas (the southern North Sea,^21^ Dutch North Sea,^21−23^ Belgian part of the North Sea,^21^ and the UK^24,25^) and specific impacts (artificial reefs,^21,24^ bottom-trawling avoidance,^25^ and noise^22^). To better quantify the impacts on marine biodiversity, a more comprehensive assessment method is required, which takes into account the long-term cumulative effects of different stressors on marine communities instead of individual species.^9^

Life cycle assessment (LCA) is a method to quantify environmental impacts over the life cycle of products. It has been used to assess the environmental impacts of electricity production from OWE on a global scale, focusing on impact categories such as climate change, marine ecotoxicity, and marine eutrophication.^26^ However, life cycle impact assessment (LCIA) methods to assess effects on marine biodiversity are still at an early stage of development and much less mature than LCIA methods for terrestrial and freshwater biodiversity.^27^ Nonetheless, LCIA methods that quantify seabed disturbance impacts on ecosystem quality have been developed. One method takes into account the change in seabed type and how long this change lasts.^28^ Another LCIA method enables assessing the impacts of noise pollution on cetaceans.^29^ Both are relevant to OWE development, but they are based on different taxa and different reference states, which do not allow for a comprehensive assessment on their own. Other LCIA methods with a focus on marine biodiversity, such as for ocean acidification^30^ or plastic debris entanglement,^31^ do not cover impact categories that are at the core of the impacts expected from OWE development, such as habitat change. Further, prior studies have mainly used hypothetical frameworks due to incomplete knowledge of the environmental mechanisms (e.g., theoretical benthic response and recovery times) and a lack of empirical data. Characterization factors (CFs) within LCIA that consider multiple relevant impacts in a consistent manner, including also positive impacts (e.g., artificial reef effect) and potential indirect impacts (e.g., trawling avoidance), are still missing.

This study aims to assess the macrobenthic (infaunal and epifaunal benthic organisms >1 mm, living within or on the seabed, respectively) biodiversity changes from artificial reefs, seabed occupation, and trawling avoidance caused by the OWF operation. The benthic biodiversity changes on hard substrates and soft sediment in different effect distances were estimated and translated into CFs. These OWE-specific CFs were derived and integrated into the LCIA framework to assess the OWE development impacts in the North Sea. This study provides a stepping stone toward a better understanding of marine biodiversity change caused by OWE development and a new perspective on sustainable OWF management.

## 2 Materials and Methods

### 2.1 Scope and Study Area

**Figure 1:**
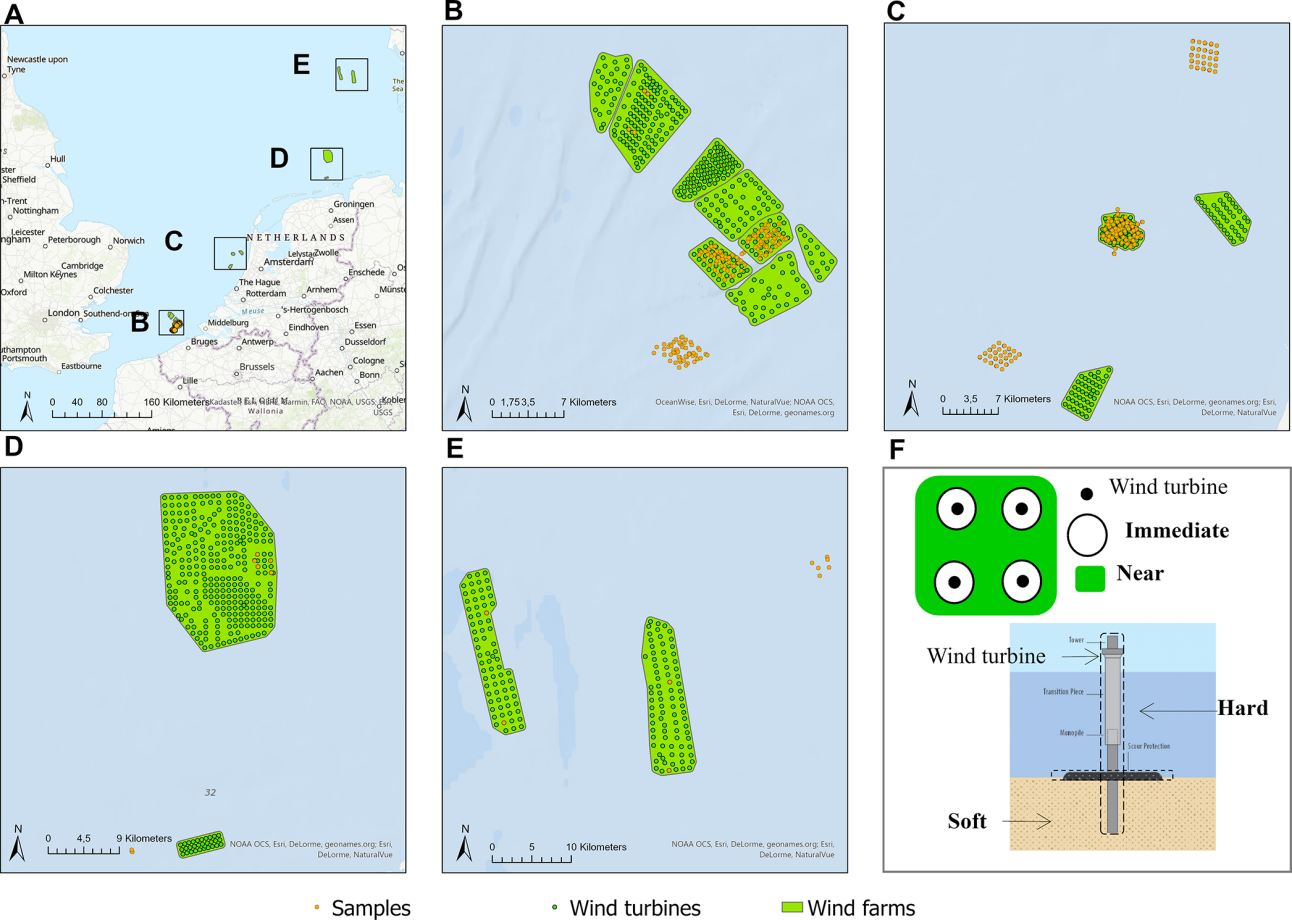
Overview of studied offshore wind farms (green areas) with turbine foundations (green dots) and sample locations (orange dots). A: the North Sea; B: Belgium; C: the Netherlands; D: Germany; E: Denmark; F: an illustration figure of effect locations (Immediate and Near) and substrate type (Hard and Soft). The maps were drawn using ArcGIS Pro, and the base map is the world topographic map from Esri (http://www.arcgis.com/home/item.html?id=30e5fe3149c34df1ba922e6f5bbf808f).

This paper develops CFs to calculate the impacts on biodiversity changes per turbine, per MW, and per total installed capacity (fleet) across the assumed 25 years of OWF operation.^26^ Biodiversity was represented by the commonly used indicators of species richness and abundance. The impacts from installation and decommissioning were excluded as they are likely temporary (several months or years) and localized when compared to impacts from operation.^35^ We considered three main interventions from OWF operations on macrobenthic communities, i.e., seabed occupation, artificial reefs, and trawling avoidance. The macrobenthos play an essential role in marine ecosystem functioning by degrading organic matter and transferring energy to higher levels in the marine food web,^32^ acting as a food source for demersal fish species and crustaceans.^33,34^ Due to data scarcity, other communities were out of the scope of this study. We used both sediment infauna and hard substrate epifauna data from OWFs and their control sites in Denmark, Germany, the Netherlands, and Belgium (Figure 1). Samples from one research platform (i.e., BeoFino), which was placed in German waters to study impacts before any OWF had been installed, were also used (Table 1). Sample data in other countries with OWE development were either not found or inaccessible (e.g., the UK). The studied OWFs, control sites, and the research platform have similar environmental conditions as they are located in the North Sea ecoregion with similar habitat types, i.e., offshore circalittoral sand or muddy patches. All wind farms have fix-bottom-based foundations (monopile, jacket, or gravity-based) and are located in shallow waters (<50 m water depth).

**Table 1:** Sample Data Overview, with Columns for the Country Offshore Wind Farm Belongs to (Country), Offshore Wind Farm Name (Wind farm), Maximal Installation Age (Age max), Samples in Different Effect Locations and Substrate Types (Immediate-Hard, Near-Hard, Immediate-Soft, Near-Soft, and Outside-Soft), and Data Source

country	wind farm	age max	immediate-hard	near-hard	immediate-soft	near-soft	outside-soft	data source
Belgium	Belwind	10	64	0	0	0	0	(43−45)
Belgium	C-power	11	21	124	14	193	152	(43, 46−48)
Denmark	Horn Rev 1	3	289	504	0	0	0	(43, 49, 50)
Germany	α Ventus	4	216	0	0	0	0	(43, 51, 52)
Germany	BeoFino	5	218	0	0	0	0	(43, 53−55)
Germany	DanTysk	6	36	0	0	0	0	(43, 50)
The Netherlands	Prinses Amalia	7	79	0	25	72	109	(23, 43, 56, 57)
total			923	628	39	265	261	

### 2.2 Sample Preprocessing

Samples were taken both on hard substrates by divers scraping fauna from the OWF/research platform foundations and from the scour protection rocks, or by collecting smaller rocks as a whole, and from soft sediment by using box cores and Van Veen grabs. Note that soft sediment data was only available in two wind farms (C-Power and Princes Amalia). For more information on the sampling method, we refer to Coolen et al.^21,42^ Organisms were identified to the species level. Sample coordinates, sample date, sample type (hard substrate or soft sediment), and sample size were registered for each OWF/research platform (Table 1). Installation age was calculated based on the time since installation and rounded to the nearest calendar year. We did not take into account that wind turbines were constructed gradually over time in a wind farm. Temporal distribution of the samples and basic information of OWFs, e.g., status of OWFs and the number of turbines, are shown in Table S1. Samples collected before installation were excluded since insufficient data was available from the different data sets.

### 2.3 Sample Geo-Processing

To distinguish different OWE interventions (i.e., seabed occupation, artificial reefs, and trawling avoidance), samples were categorized into two substrate types (STs), i.e., Hard (hard substrate) and Soft (soft sediment). Samples were further classified into three effect locations (ELs) by distance, i.e., Outside (distance to the nearest wind farm >500 m), Near (distance to the nearest wind farm ≤500 m and distance to the nearest turbine ≥250 m), and Immediate (distance to the nearest turbine <250 m) (Figure 1). The distances to the nearest farm and the nearest turbine were calculated by ArcGIS Pro toolbox “Geoprocessing”–“Near.” We used wind turbine coordinates from the global OWF database.^36^ We defined a convex hull of each OWF, i.e., the smallest convex polygon that contains all wind turbines of a farm, and added a buffer (500 m) by using ArcGIS Pro to delineate a 500 m security zone around a farm, within which bottom trawling is generally prohibited.^37^ We assumed that seabed occupation and artificial reefs impact macrobenthos within 250 m diameter of the wind turbine foundation.^38^ In summary, our data set covers between 39 and 923 samples for each combination of substrate type and effect location (Table 1).

### 2.4 Biodiversity Model Fit and Estimation

Sample data (covering up to 11 years of turbine life) was used to fit a generalized linear mixed model (GLMM) and then estimate the biodiversity values from 12 to 25 years after installation.
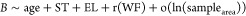
1where biodiversity (*B*) is represented by species richness and abundance. A Poisson distribution with an ln link was used in the GLMM. Installation age (age), substrate type (ST), and effect location (EL) were considered key parameters for biodiversity and included as fixed effects in the model. We added the wind farm (WF) to the model as a random effect, i.e., r(WF), as samples taken in the same year, substrate type, or effect location could be affected by wind farm variation in environmental conditions. The offset term o(ln(sample_area_)) was used to adjust the richness for different spatial extent within sampled surface area, since the richness and the sampled area relation is nonlinear.^39^ Abundance was directly standardized to a sampled area of 1 m^2^. The assumptions of homogeneity of variance, normality, and variable collinearity were checked, and a diagnosis report can be found in the Supporting Information (SI). Additionally, a model validation was conducted, and prediction error-based indicators are reported in the SI. R version 4.0.1^40^ and RStudio version 2022.2.1.461^41^ were used for the data analysis.

Species diversity from five oil and gas platforms (Outside-Hard) (Table S3), operated by ENGIE Exploration & Production Nederland B.V. (ENGIE), was calculated as a sanity check for richness and abundance in Immediate-Hard. The oil and gas industry has a long history of offshore development in the North Sea and implements a similar foundation structure as offshore wind farms. Although with considerable uncertainty in quantification and with annual fluctuation, the evolution of biodiversity from 12 to 25 years after OWF installation was assumed to be similar to observed old oil and gas platforms. The average values of richness and abundance from the installation of these platforms (in the period 1972–1999) to 2014–2015 were calculated. The biodiversity values from the GLMM were cut off and corrected if they went beyond the range of those in the oil and gas platforms.

### 2.5 Characterization Factor (CF) Development

We developed CFs based on the difference in biodiversity (*B*) between the OWE intervention (seabed occupation, artificial reefs, or trawling avoidance) and the associated reference state (RS), which is a state that represents today’s state but without such intervention. The average biodiversity values (richness and abundance) in different ELs and STs from installation to 25 years of operation were used to reflect the benthic response to an intervention.
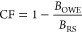
2The proposed CFs express the potentially disappeared fraction of species (PDF) or abundance (PDF_A_). Therefore, positive CF (ranges from 0 to 1) reflects a relative loss of species or decrease in abundance. Negative CF (any negative number) represents a relative gain of species or an increase in abundance. Different ELs and STs result in three CFs in line with three interventions, i.e., artificial reefs (CF_AR_), seabed occupation (CF_SO_), and trawling avoidance (CF_TA_). Artificial reefs and seabed occupation are relatively local (Immediate). Trawling avoidance impacts larger areas (both Immediate and Near). More specifically, CF_AR_, CF_SO_, and CF_TA_ were quantified based on Immediate-Hard, Immediate-Soft, and Near-Soft, respectively. CF_TA_ excluded Immediate-Soft as seabed occupation also affects the Immediate-Soft. The RS for CF_AR_ and CF_SO_ was set to Near-Soft to isolate their effects, as trawling avoidance affects both the Immediate and Near effect locations. The RS for CF_TA_ was set to Outside-Soft, which represents the natural variation of biodiversity.

### 2.6 Inventory Analysis and Impact Assessment

To assess the impacts (biodiversity changes) of each intervention and discuss the cumulative impacts, our CFs were further combined with associated affected areas along the OWF operation time in line with three functional units (per turbine, per MW, and fleet). Artificial reefs and seabed occupation have the same size of impact areas (i.e., Immediate, 0.0625 km^2^ per turbine), although they impact different STs (i.e., Hard and Soft, respectively). Trawling avoidance has a larger impact area (i.e., Near), and its impact area per turbine was calculated based on the size of the wind farm with a 500 m buffer divided by the wind turbine count. The size of each wind farm with the buffer was calculated by ArcGIS Pro toolbox “Geoprocessing”–“Calculate Geometry Attributes.” The impact assessment results per turbine were further converted to per MW and per fleet. Lifetime extension and turbine size growth are expected in the future, which control the affected time and area, respectively. The average individual turbine capacity is likely to reach 20 MW (compared to ∼6 MW in 2020), and lifetime will increase to 30 years in 2050.^26^ The impacts per MW were calculated based on impacts per turbine divided by average individual turbine capacity. A linear regression was used to model the turbine capacity projection to 2040^26^ and extended to 2050 (Table S4). The area affected by artificial reef and seabed occupation effects is assumed to be proportional to the foundation size. The area subjected to trawling avoidance depends on the OWF size (further details in 2.6 in SI). In terms of fleet impacts, 300 GW of installed OWE capacity is targeted in the North Sea by 2050.^2^ Considering the turbine size development, the North Sea will require 15,000–50,000 wind turbines by 2050.

### 2.7 Sensitivity Analysis

Impact areas may change depending on the ecological context and reference state.^42^ To carefully address different spatial scales in the evaluation of three interventions, we conducted a sensitivity analysis by altering the thresholds used for the EL categorization by 50%, i.e., ±50% of 500 m buffer around the wind farm and ±50% of 250 m distance from the turbine. An additional sensitivity analysis was conducted by comparing results of only considering the observations (average biodiversity values through 11) and max yearly average within the data range (observed at age 11) to the whole time series (with estimation until age 25). Further, results of randomly leaving out 20% of samples were compared to those with all samples.

## 3 Results

### 3.1 Biodiversity Evolution in Different Effect Locations (ELs) and Substrate Types (STs)

**Figure 2:**
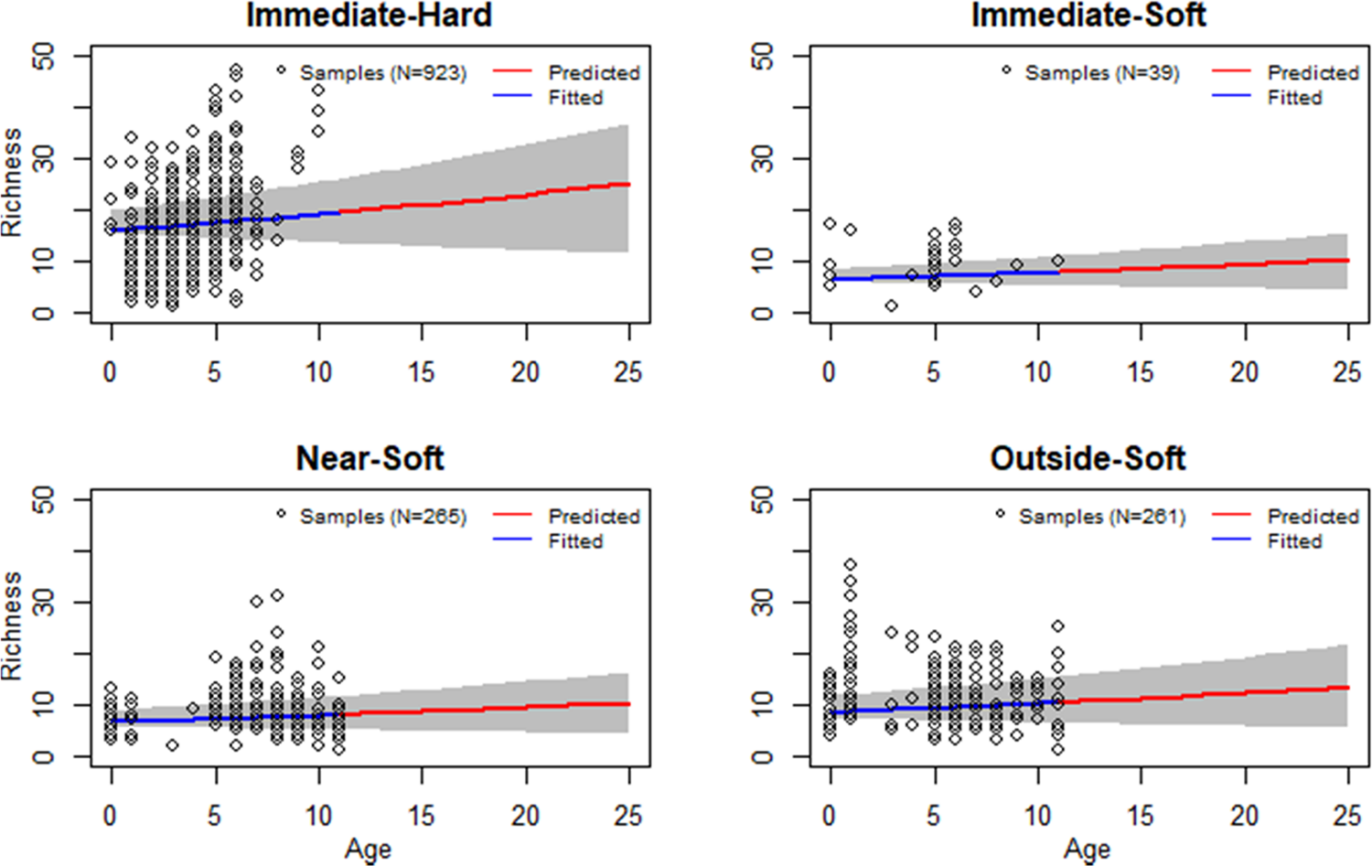
Species richness evolution from installation to 25 years afterward. The gray shaded area shows the 95% confidence interval.

**Figure 3:**
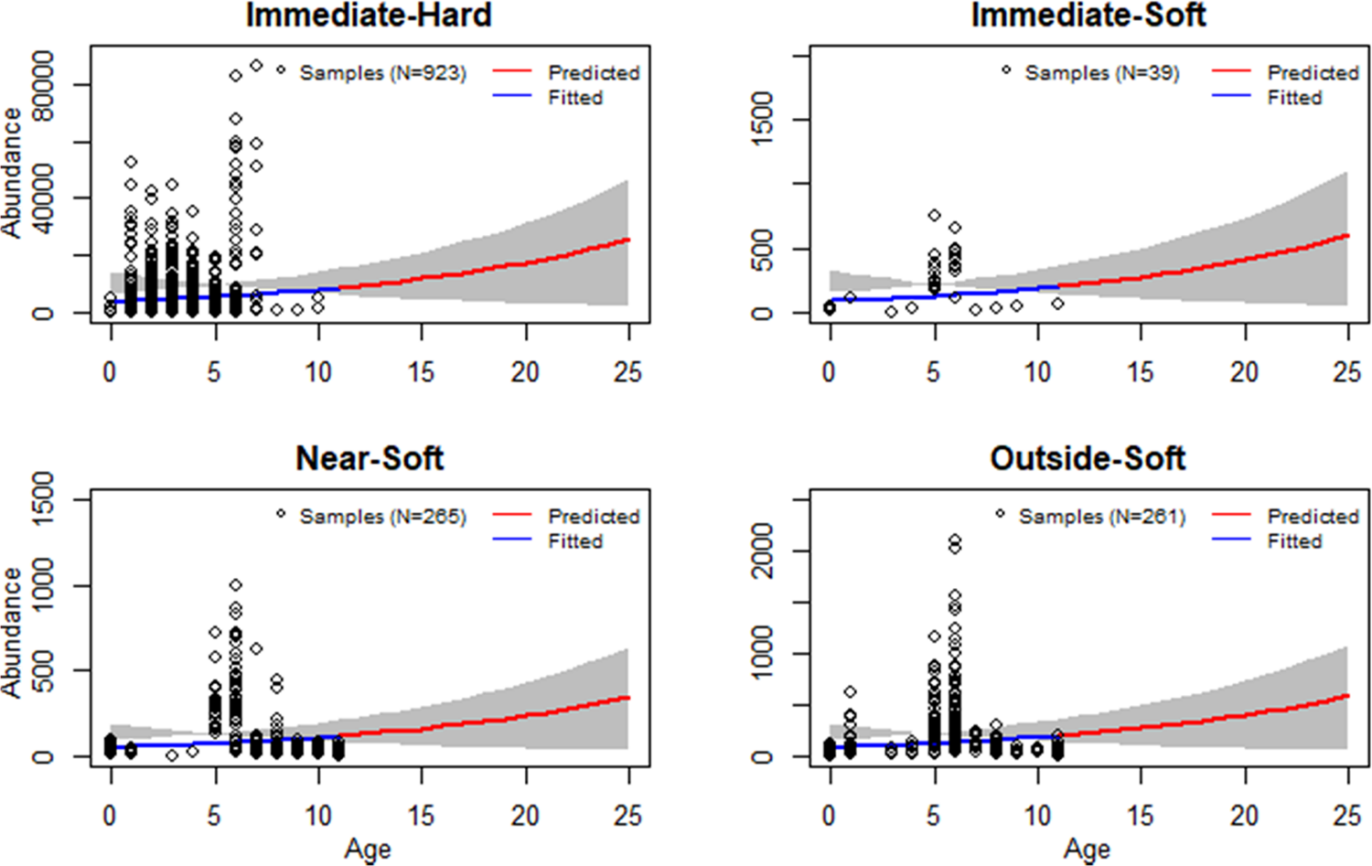
Species abundance (individuals/m^2^) evolution from installation to 25 years afterward. The gray shaded area shows the 95% confidence interval. Note that the subplots are not directly comparable because the *y*-axis limits differ.

Species richness and abundance on hard substrates are greatly higher than on soft sediment and will increase over time since OWF construction (Table S6, Figures 2 and 3). Species richness in Immediate-Hard will increase from ∼17 species per 0.01 m^2^ one year after installation to ∼23 species per 0.01 m^2^ at the end of the OWE turbine lifetime (Figure 2). Abundance in Immediate-Hard at the end of turbine lifetime is expected to quadruple compared to one year after installation (Figure 3) as the community structure gradually changes after the switch from soft to hard substrate. Sanity check results (Table S3) show that the species richness and abundance values in Immediate-Hard are within the range of those oil and gas platforms (Outside-Hard).

Species richness in all Soft categories is below 13 per 0.01 m^2^ along the turbine lifetime (Figure 2). Species richness in the Immediate-Soft is slightly lower than in the Near-Soft and much lower than in the Outside-Soft (Figure 2 and Table S6). Spatially, species richness decreases toward wind turbines. Species abundance in the Immediate-Soft is higher and slightly higher than that in the Near-Soft and Outside-Soft, respectively (Figure 3 and Table S6).

### 3.2 Characterization Factors

**Figure 4:**
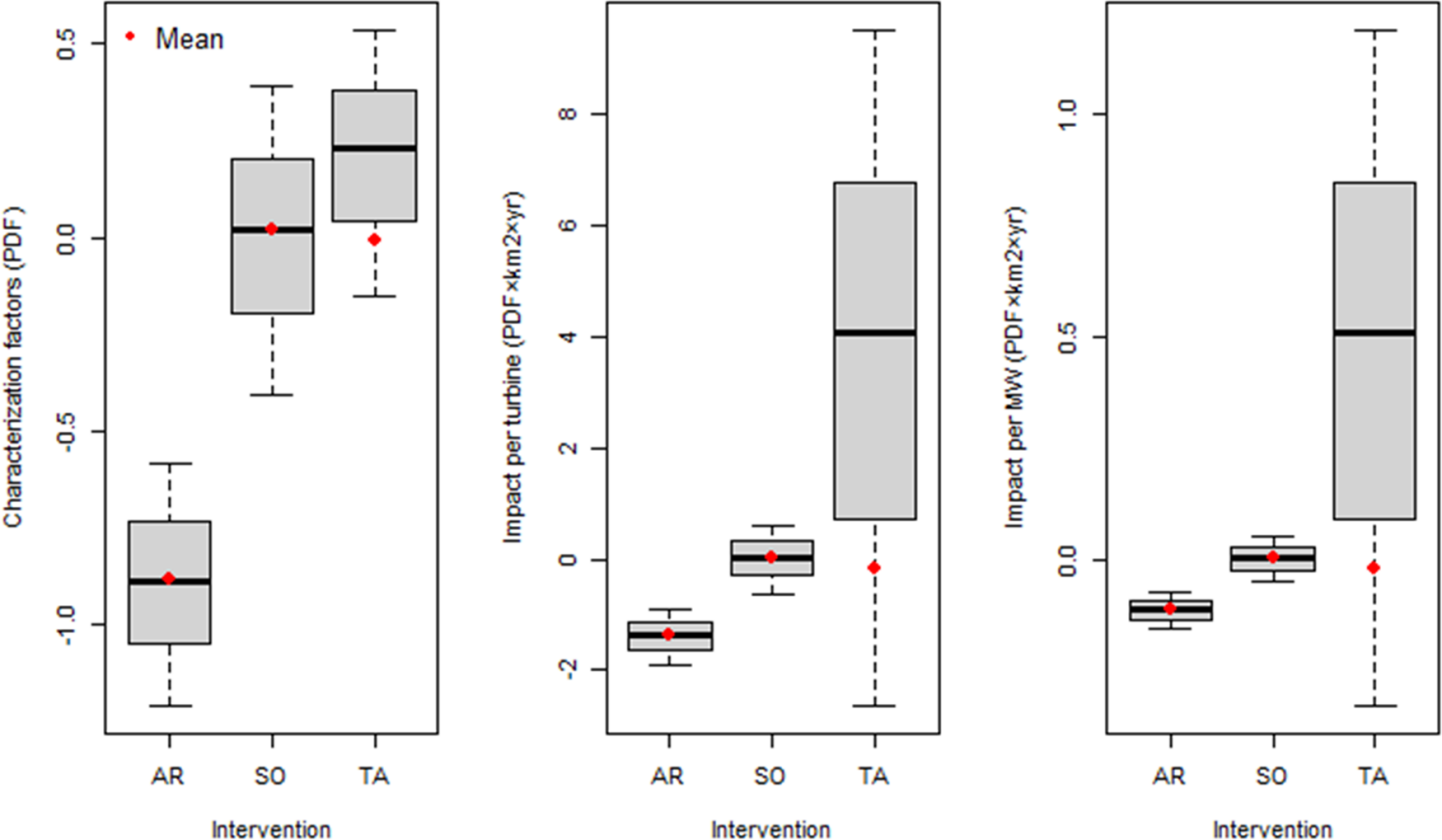
Characterization factors and impact assessment results for species richness. AR: artificial reefs, SO: seabed occupation, and TA: trawling avoidance. The average values (red points) are used for CFs and impact assessment results in this study. Note that the average of negative values is applied for TA.

**Figure 5:**
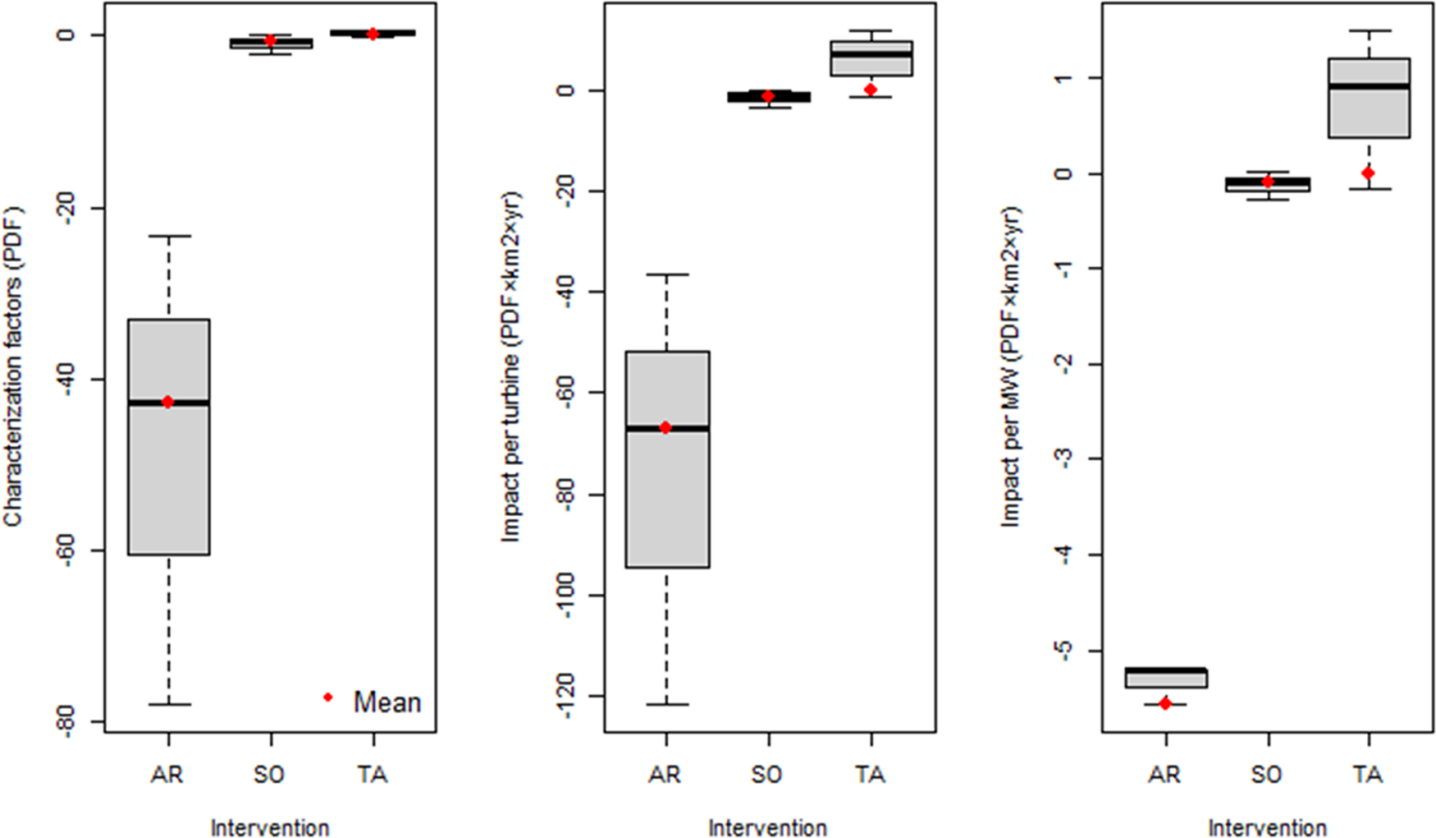
Characterization factors and impact assessment results for species abundance. AR: artificial reefs, SO: seabed occupation, and TA: trawling avoidance. The average values (red points) of CF and impact assessment results are used in this study. Note that the average of negative values is applied for TA.

We apply the developed CFs to biodiversity changes associated with three OWE interventions (i.e., artificial reefs, seabed occupation, and trawling avoidance) in characterization perspectives:

(1) Artificial reef: Marine benthic biodiversity on hard substrates will significantly increase due to the artificial reefs. CFs for artificial reefs (CF_AR_) show −0.88 PDF and −42.87 PDF_A_ (Table S6, Figures 4 and 5), which indicates a doubling of species richness and an increase by two orders of magnitude for abundance on hard substrates.
(2) Seabed occupation: CFs for seabed occupation CF_SO_ range from −0.41 to 0.39 PDF and from −2.14 to 0.05 PDF_A_ (Table S6, Figures 4 and 5). So, for the effect locations and substrate types for which we assume seabed occupation effects, both positive and negative impacts can occur.
(3) Trawling avoidance: Our results do not reflect the trawling avoidance benefits from the OWF. Nonetheless, we apply the average of negative values as the CF results for trawling avoidance, i.e., −0.10–0.00 PDF and −0.01–0.00 PDF_A_ (Table S6, Figures 4 and 5).

### 3.3 Inventory Analysis and Impact Assessment

The impact area for artificial reef and seabed occupation effects is 0.06 km^2^ per turbine. 0.71 km^2^ of area per turbine is supposed to be affected by trawling avoidance based on the studied OWFs. Impacts of artificial reefs on species richness and abundance are −1.38 PDF km^2^·yr and −66.99 PDF_A_·km^2^·yr, respectively. Impacts of seabed occupation are approximately 1.35 (98%) PDF·km^2^·yr and 65.86 (98%) PDF_A_·km^2^·yr lower than the absolute values of artificial reef effect, respectively. Trawling avoidance effects range from −1.89 to 9.31 PDF·km^2^·yr and from −1.41 to 11.98 PDF_A_·km^2^·yr (Figures 4 and 5). In summary, one turbine has net biodiversity gains for benthic species (based on the average of negative values of CF_TA_), i.e., −1.34 PDF· km^2^·yr and −65.64 PDF_A_·km^2^·yr.

As wind turbine sizes grow, artificial reef and seabed occupation effects could slightly decrease per MW since large turbines have smaller affected areas per MW than smaller turbines. However, the trawling avoidance-affected areas per MW could greatly increase when turbine size and, thus, distance between foundations expand (Table S4). Wind turbine lifetime extension will increase all impacts discussed in this study. Impact assessment results per fleet show that impacts from −1.01 × 10^5^ to −5.71 × 10^4^ PDF· km^2^·yr and from −3.72 × 10^6^ to −2.61 × 10^6^ PDF_A_·km^2^·yr are expected in the North Sea from 2020 to 2050.

### 3.4 Sensitivity Analysis

Our results demonstrate that ±50% of 500 m buffer is responsible for only slight changes in CFs. However, ±50% of 250 m will considerably affect the CF results. There is no substantial difference between CF results when considering estimated values and without considering estimated values (average values through age 11 or max yearly average within the data range). The species richness and abundance in all categories increase over time, which shows the robustness of CF results. Randomly leaving out 20% of samples hardly affects the CF results (Table S7).

## 4 Discussion

Knowledge impacts of OWE development on marine biodiversity are still limited. To our knowledge, this is one of the first studies to explore patterns of change in marine biodiversity in different ELs and STs. This study takes one step further by extrapolating marine biodiversity in time/area and integrating this knowledge into a broader perspective by developing empirical CFs that can be used in LCA studies. Three developed CFs enable us to separately assess three interventions by the OWE development, i.e., artificial reefs, seabed occupation, and trawling avoidance. Although the interventions considered in this study act simultaneously during OWF operation, they affect different ELs and STs with different benthic communities. Adopting separate CFs broadens the application of CFs, and each CF could be used for similar interventions by other marine activities (e.g., CF_SO_ is also relevant to cable laying). The developed CFs provide a stepping stone toward a better representation of biodiversity in LCA studies. Although the simulation goes beyond observed data, our sensitivity analysis verifies the feasibility of considering the 25-year operation time (Table S7). Scenarios will be developed to show different trajectories of future biodiversity evolution. Future research should extend such monitoring time series and consider more site conditions. For instance, site monitoring should happen much before the OWF construction and continue throughout the OWE lifetime. Monitoring should also include (future) sites outside the current developed areas. Moreover, more monitoring efforts should be done in soft sediment, which enables us to better understand specific interactions with the local marine environment and take steps to avoid or minimize negative impacts. This could be achieved by parameterizing our CFs by considering more site-specific environmental parameters (e.g., more detailed substrate types, water depth, water temperature, and seasonal patterns). Collaborative efforts from industry, academia, and government are needed to leverage more knowledge, data, and resources.^58^

### 4.1 Interpretation and Limitations of CFs

The enhancement of forage bases and piscivorous predators by the artificial reefs could explain the increase in biodiversity on hard substrates.^59^ However, artificial reefs will also attract new species (nonindigenous species). The positive CF results for seabed occupation might be explained by a substantially higher mortality/migration of certain native benthic species within the immediate zone than in surrounding areas, but the impact is short-term (1 year), especially in sandy sediments that are poor in infauna diversity.^35^ The increased biodiversity in the nearby hard substrates can partly spill over to the soft sediments, as deposition in the form of fecal pellets expelled by filtering epifauna leads to an increase in organic matter.^60^ This could also be considered an effect of artificial reefs, although the CFs for the artificial reefs in our study refer to that on hard substrate. Consequently, the biodiversity of the original soft sediment fauna decreases slightly, but new (other) species come into the area, so the species richness and abundance increase.^46^

A limitation of our work is that, in line with the current LCIA methods for biodiversity, we took into account species richness and abundance only. Future research is required to understand the ecological effects^44^ of biodiversity change. This could be done by expanding the analysis to a community level, looking into the potential modification of community structure. The slight decreases in species richness and an increase of abundance in the immediate soft sediment might lead to a less healthy community in certain directions around a wind turbine.^61^ The introduction of artificial reefs might trigger an increase in opportunistic species density.^62^ We acknowledge that in general, this statement might be true for areas hosting rare species, such as gravel beds. For the Belgian and Dutch OWFs, the original community consisted of opportunistic species. Macrobenthos in the species-poor sandbanks recovered quickly after OWF construction, and no rare species were lost.^62^

It is difficult to demonstrate a trawling avoidance effect since our soft sediment data was only collected in two OWFs (C-Power and Princes Amalia) in relatively short time frames. Another issue is that there are currently no good control sites. Natural spatial variability complicates the detection of a trawling avoidance effect. The biodiversity values in the reference area outside of Belgian wind farms were already higher than within the wind farms before OWF installation.^35^ The locations studied were geographically separated, and future research needs to include natural reefs. A larger sample size through more wind farm studies in a longer temporal range is also required to properly test this effect. OWFs are currently closed to trawl fisheries in the North Sea.^63^ In Germany, the OWE development is assigned high priority to sea use and adheres to strict safety regulations.^64^ Bottom-disturbing activities, like anchoring or dragging of fishing gear, are forbidden within the Dutch and Belgian OWF safety zones.^65^ However, the trawling avoidance by the OWFs may cause more intensity of trawling in areas outside OWFs. When wind turbines become bigger with larger spacing between turbines, ships would be allowed to pass through OWFs,^65^ although trawling is still forbidden. The trawling free zones within OWFs have been considered multiuse options to better use ocean space for energy generation.^66,67^ For example, integration of floating photovoltaics into OWFs will increase power conversion efficiency and seems to have insignificant effects on fish populations, although long-term environmental impacts, e.g., shading effects, remain unexplored.^68^

### 4.2 Effect of Geography and Range

The results presented in this study may not be suitable to be applied in other ecoregions as different habitat types may have different patterns of change in benthic communities. Results based on 3 years of sample data from the Block Island wind farm in the US^69^ (Table S8) show major differences in species richness and abundance compared to the North Sea. Further onsite sampling efforts for OWFs in other ecoregions will benefit a better understanding of biodiversity change on a larger scale. However, the proposed CFs could still be applied in other ecoregions as similar effects might be expected to occur elsewhere. In addition, at the regional scale, this study focuses on the North Sea, which is one of the OWE hotspots.

Artificial reefs and seabed occupation can have larger effect areas than we considered in this study. However, to what extent the effects will impact benthic communities outside the diameter of the wind turbine, in soft sediment, or even the pelagic compartment remains unknown. It is also challenging to separate anthropogenic impacts and natural variability in the OWE developed areas.^70^ Long-lasting monitoring in Immediate and Near, at the surface and near the bottom of the wind turbine is required to gain a better understanding of how effects radiate outward from the wind turbine and how far. More samples in control/reference sites also allow for better sampling designs. Further, monitoring efforts should move toward information-rich data collection (e.g., distilling site-specific and ecological responses across areas and species), thus allowing a broader scale of interpretation of OWE interventions.^7^ Monitoring methods like environmental DNA (eDNA) metabarcoding with a higher chance of detecting species could be an alternative to the current time-consuming and costly routine biomonitoring.^71^

### 4.3 Interventions in Other Life Cycle Stages

The macrobenthic biodiversity can be affected and/or modified by OWF installation and decommissioning,^9^ although these effects are likely to be more localized and shorter when compared to that of OWF operation. Processes during installation, e.g., pile driving, dredging, and smoothing, create noise and vibration that impact seabed habitats.^10^ This study did not take into account that wind turbines were constructed gradually over time in a wind farm, and construction may continue after the first turbines already started operation. The OWF installation time is highly uncertain and depends on, for instance, OWF size, foundation type, site condition, and equipment availability.^72^ ∼20% of samples were collected at an installation age of 0 or 1, which might affect our results as some impacts might come specifically from installation. Effects from decommissioning are still poorly known as only a limited number of OWE projects have been decommissioned. The complete removal would be the opposite process of installation,^18^ but it is controversial due to potential impacts on the ecosystem. Proposals for alternative uses of hard substrates from the OWE infrastructure, e.g., renewables-to-reefs,^73^ are expected. Partial removal (cutting part of the foundation and leaving the rest in situ) will create lesser disruptions to the colonizing benthic communities around the foundations.^74^ There are also social and engineering aspects to be considered in removing a foundation. Future studies should consider more interventions along the OWF life cycle and assess the cumulative effects. Some lessons could be learned from the oil and gas industry.^37^

### 4.4 Technology

Different OWE foundation technologies will create different artificial reefs in terms of size and materials,^75^ which affect the success and degree of habitat creation and use.^76^ Foundation technologies also determine the installation and decommissioning processes,^77^ which result in varied stressors on the seabed with different impacts on benthic communities (Table S9). Future work is needed to include more foundation types, especially floating foundations, which are assembled on land and then connected to mooring cables. Although anchor installation may involve pile driving and mooring cables that produce noise during operation, the floating foundations are expected to cause less vibration and noise than fix-bottom-based foundations. Turbine (i.e., nacelle, rotor, and tower) and transmission (e.g., cables, transformers, and substations) technologies are expected to have minor impacts with slight variations on benthic species.

## Supporting Information Available

More detailed documentation of methods; model validation results; Q–Q plot of the species richness model for a normal distribution of the residuals; model residuals against the fitted values for the species richness model; inventory analysis and impact assessment; sensitivity analysis results for characterization factors (PDF)
