# Supplementary material for: Offshore Wind Energy and Marine Biodiversity in the North Sea: Life Cycle Impact Assessment for Benthic Communities
Source: Environ Sci Technol. 2023 Apr 14;57(16):6455–64. doi: 10.1021/acs.est.2c07797 (PMC10134491; doi:10.1021/acs.est.2c07797)
Supplement: Supplementary file 1 — es2c07797_si_001.pdf [file es2c07797_si_001.pdf]

Supporting Information

Offshore wind energy and marine biodiversity in the North Sea: life cycle impact assessment for benthic communities

Chen Li[1]*, Joop W.P. Coolen[2,3], Laura Scherer[1], José M. Mogollón[1], Ulrike Braeckman[4,5], Jan Vanaverbeke[5], Arnold Tukker[1,6], Bernhard Steubing[1]

[1] Institute of Environmental Sciences (CML), Leiden University, P.O. Box 9518, 2300 RA Leiden, the Netherlands
[2] Wageningen Marine Research, P.O. Box 57, 1780 AB Den Helder, The Netherlands
[3] Wageningen University, Aquatic Ecology and Water Quality Management Group, Droevendaalsesteeg 3a, 6708 PD Wageningen, The Netherlands
[4] Ghent University, Marine Biology Research Group (MARBIOL), Krijgslaan 281, 9000, Ghent, Belgium
[5] Royal Belgian Institute for Natural Science, Operational Directorate Natural Environment, Marine Ecology and Management, Vautierstraat 29, 1000, Brussels, Belgium
[6] Netherlands Organization for Applied Scientific Research, P.O. Box 96800, 2509 JE Den Haag, the Netherlands

* Corresponding author: c.li@cml.leidenuniv.nl

Summary information:

The number of pages: 7

Tables: 9

Figures: 4

2.1 Scope and study area

Table S1: Sample data overview, with columns for offshore wind farm name (Wind farm), number of turbines in the wind farm (# Turbines), wind farm status (Status), and installation age (Age).

Wind farm	# Turbines	Status	Age											
			0	1	2	3	4	5	6	7	8	9	10	11
Belwind	55	In operation	4	12	18	9	6	3	3	0	3	3	3	0
C-power	54	In operation	56	60	31	34	15	31	51	51	59	44	36	36
Horn Rev 1	80	In operation	0	257	268	268	0	0	0	0	0	0	0	0
Alpha Ventus	12	In operation	0	36	72	72	36	0	0	0	0	0	0	0
BeoFino[1]	-	-	0	5	32	63	78	40	0	0	0	0	0	0
DanTysk	80	In operation	0	0	0	0	0	18	18	0	0	0	0	0
Prinses Amalia	60	In operation	0	0	0	0	10	132	133	10	0	0	0	0
Total			60	370	421	446	145	224	205	61	62	47	39	36

2.4 Biodiversity model fit and prediction

The Q-Q plot was used to visually check the assumption of normality. The results show that the models do not raise significant concern with normality of residuals (**Figures S1** and **S2**), also given that linear mixed-effect models are quite robust to violations of the assumption [3].

The model residuals were plotted against the fitted values to check homogeneity of variance. The results suggest that homogeneity of variance can be assumed (**Figures S3** and **S4**).

The variance inflation factor [4] was used to check the possible multicollinearity between predictors. The variance inflation factors were <2.5 for all predictors; therefore, multicollinearity was not an issue for the considered predictors [4].

The Monte Carlo cross-validation was further conducted to judge the performance of the GLMM model. The dataset used to build the GLMM is randomly divided into training set (80%) and validation set (20%). The accuracy of the GLMM is calculated through prediction error-based indicators, i.e. mean squared error (MSE), mean absolute error (MAE), mean absolute percentage error (MAPE) and coefficient of determination (R^2). The simulation was conducted 50 times and the average values were used (**Table S2**). The results indicate the high and acceptable accuracy of the GLMM for species abundance and richness, respectively.

Table S2: Model validation results.

**Species richness**				**Species abundance**			
MSE	MAE	MAPE	R^2	MSE	MAE	MAPE	R^2
27.11	4.23	0.27	42%	556192.36	1452.15	0.18	87%

[1] Research platform

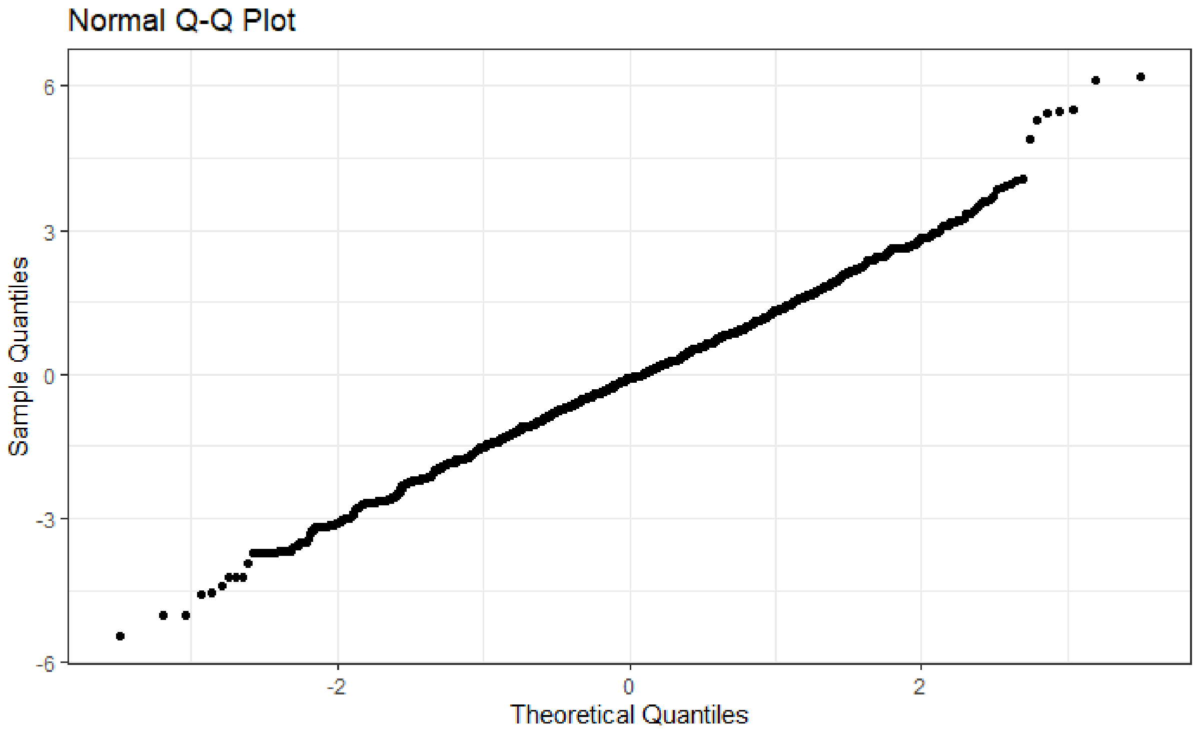

Figure S1: Q-Q plot of the species richness model for a normal distribution of the residuals.

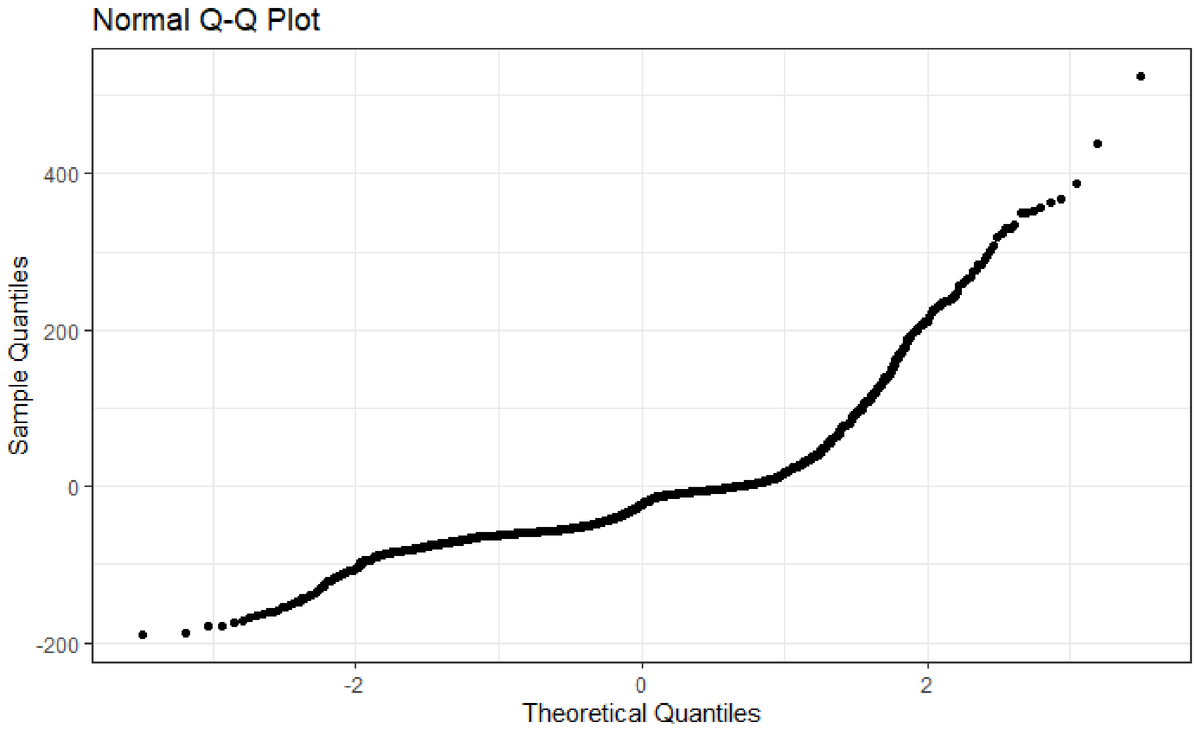

Figure S2: Q-Q plot of the species abundance model for a normal distribution of the residuals.

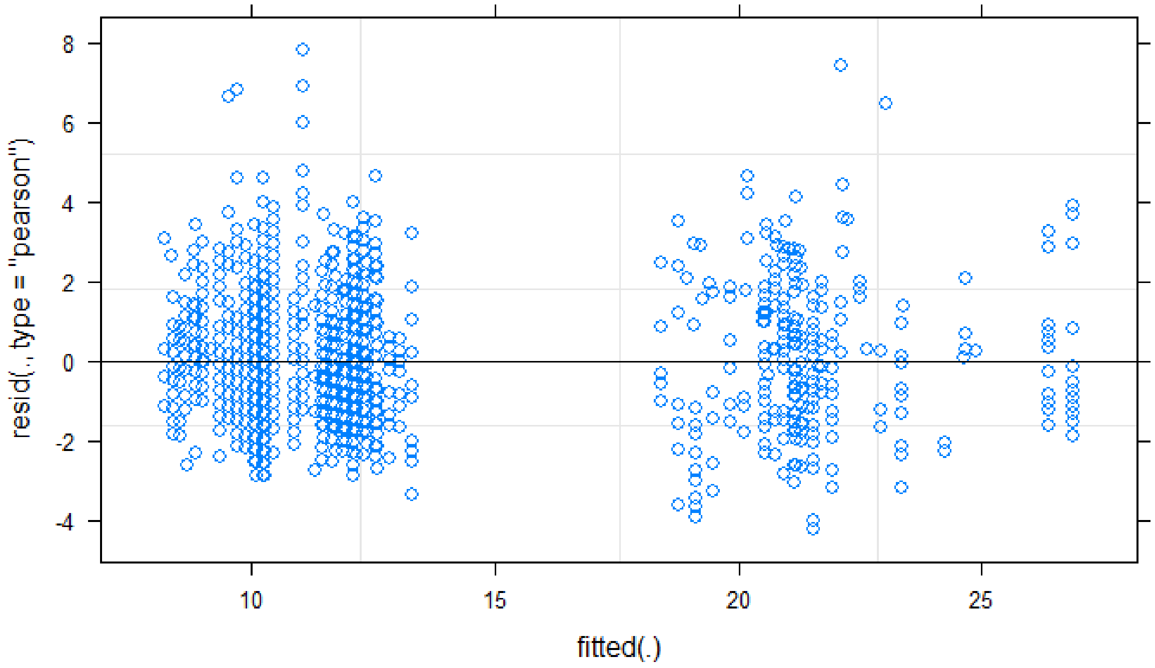

Figure S3: Model residuals against the fitted values for the species richness model.

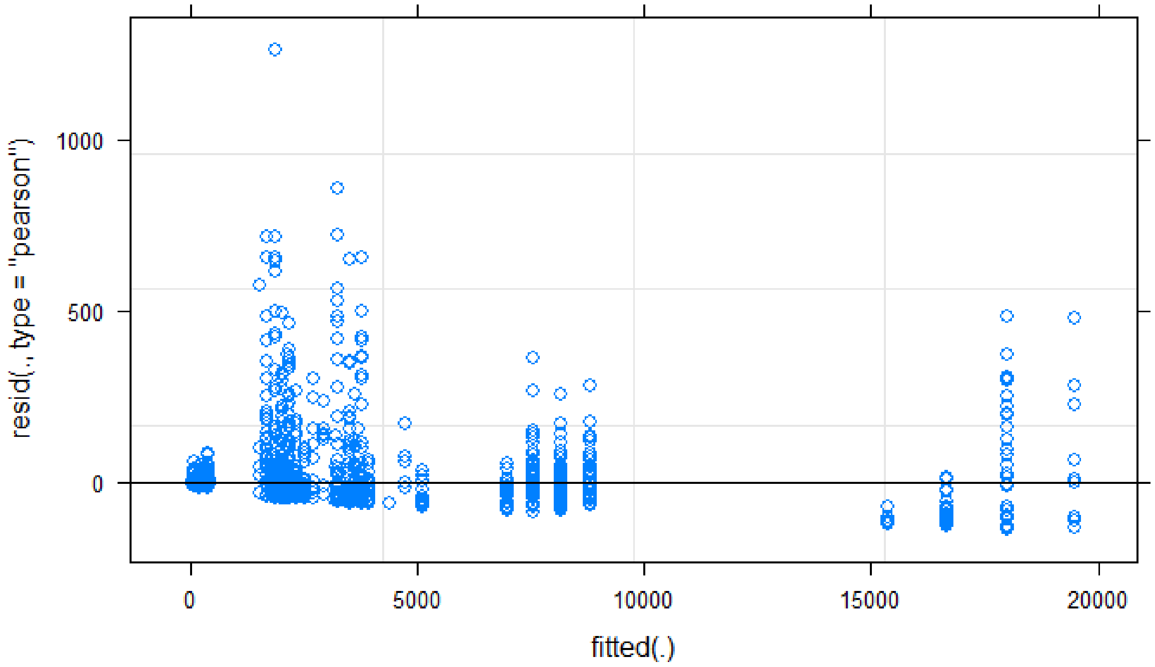

Figure S4: Model residuals against the fitted values for the species abundance model.

2.5 Characterization factor (CF) development

Table S3: Species richness and abundance of oil and gas platforms (**Outside_Hard**) (BS, D15, Halfweg, K9-A, L10-A, L10-G, L15-A, and Q13).

Station	Age max (yrs)	Richness	Abundance (ind./m²)
BS	8	27.36	10900.45
D15	17	30.76	28297.81
Halfweg	21	28.02	16166.75
K9-A	19	28.73	8552.54
L10-A	44	33.29	23926.99
L10-G	32	24.76	12450.51
L15-A	23	48.85	21866.71
Q13	2	17.33	59166.70

2.6 Inventory analysis and impact assessment

OWE foundation size was assumed to determine the affected area by artificial reef and seabed occupation effects. Foundation size is found linearly related to the turbine size [1]. We calculated affected areas based on the assumptions from Negro et al. [1] (**Table S4**). The area subjected to trawling avoidance depends on the OWF size, which is determined by the spacing between turbines and columns (**Table S4**). The estimation of spacing between turbines and columns (i.e. spatial configuration in which turbines are aligned in a wind farm) was based on rotor diameter. As turbine size grows, the spacing between turbines and the spacing between columns are expected to grow.

Table S4: Estimation of turbine size, OWF layout and impact areas.

	2020	2025	2030	2040	2050
Single turbine capacity (MW) [2]	6	8	12	15	20
Rotor diameter (m) [2]	120	150	200	250	300
Spacing between columns (km) [2]	1.44	1.80	2.40	3.00	3.60
Spacing between turbines (km) [2]	1.20	1.50	2.00	2.50	3.00
Affected areas by artificial reef (km²/turbine)	0.06	0.09	0.13	0.16	0.19
Affected areas by seabed occupation (km²/turbine)	0.06	0.09	0.13	0.16	0.19
Affected areas by trawling avoidance (km²/turbine)	0.71	1.60	2.84	4.44	6.39
Affected areas by artificial reef (km²/MW)	0.01	0.01	0.01	0.01	0.01
Affected areas by seabed occupation (km²/MW)	0.01	0.01	0.01	0.01	0.01
Affected areas by trawling avoidance (km²/MW)	0.09	0.20	0.24	0.30	0.32

3.1 Biodiversity evolution in different effect locations (ELs) and substrate types (STs)

Table S5: Summary statistics of the GLMM, with columns for estimate of independent variables (Estimate), standard error (SE), and p-value (P, <0.001 = ***, <0.05 = *), rows for installation age (Age, years since installation), effect locations (EL_Near: Near, EL_Out: Outside), substrate type (ST_Soft: Soft), adjusted R^2 (R^2) for the model, and statistical power (SP) based on 95% confidence interval. Reference level for EL: EL_Immediate. Reference level for ST: ST_Hard.

	**Species richness**			**Species abundance**		
	Estimate	SE	P	Estimate	SE	P
Age	0.02	0.00	***	0.08	0.00	***
EL_Near	0.02	0.02	0.27	-0.55	0.00	***
EL_Outside	0.28	0.03	***	-0.02	0.01	***
ST_Soft	-0.91	0.03	***	-3.76	0.00	***
R^2			39%			85%
SP			100%			100%

Table S6: Biodiversity (species richness and abundance) evolution and resulting characterization factors (CF) for artificial reefs (AR), seabed occupation (SO), and trawling avoidance (TA). LL: lower limit of 95% confidence interval; UL: upper limit of confidence interval. Note that CF_{TA} are calculated based on the average of negative values of PDF.

		Immediate-Hard	Immediate-Soft	Near-Soft	Outside-Soft	CF_{AR}	CF_{SO}	CF_{TA}
**Richness**	LL	13.23	5.04	4.97	6.29	-1.21	-0.41	-0.10
	Mean	20.17	8.09	8.26	10.70	-0.88	0.02	-0.01
	UL	27.56	11.61	12.30	16.31	-0.58	0.39	0.00
**Abundance**	LL	6325.56	146.06	84.41	141.06	-77.99	-2.14	-0.01
	Mean	11388.3	265.49	153.82	259.57	-42.87	-0.73	0.00
	UL	20502.59	482.54	280.28	477.63	-23.37	0.05	0.00

3.4 Sensitivity analysis

Table S7: Sensitivity analysis results for characterization factors.

		Default	+50% of 500 m buffer	-50% of 500 m buffer	+50% of 250 m	-50% of 250 m	Until age 11[2]	At age 12 [3]	Leave 20% samples
**Richness**	CF_{AR}	-0.88	-0.89	-0.88	-0.92	-0.88	-0.88	-0.88	-0.90
	CF_{SO}	0.02	0.02	0.02	-0.06	0.02	0.02	0.02	0.02
	CF_{TA}	-0.01	-0.01	-0.01	0.00	-0.01	-0.01	0.00	0.00
**Abundance**	CF_{AR}	-42.87	-42.88	-42.86	-39.44	-45.07	-43.55	-42.87	-42.93
	CF_{SO}	-0.73	-0.73	-0.75	-0.77	-0.77	-0.75	-0.72	-0.73
	CF_{TA}	0.00	0.00	-0.02	0.00	0.00	0.00	0.00	0.00

4.2 Effect of geography and range

Table S8: Species richness and abundance of Block Island wind farm (**Immediate_Hard**) [5].

Age	Richness	Abundance (ind./m^2)
1	19.52	151.41
2	22.95	572.50
3	24.86	464.37

4.4 Technology

Table S9: Installation of different types of foundations [2].

Foundation type	Foundation name	Suitable water depth	Suitable seabed type	Stressor	Foundation size (m^2) [6]
I	Gravity-Base High-Rise Pile Cap	Shallow (normally	Compacted clay, sandy soil,	Substrate clearance	1195

[2] Only considering observations (i.e. until age 11).

[3] Max modelled average within the data range (i.e. at age 12).

		<10m, up to 30m)	and rock	Substrate replacement<br>Driving pile<br>Scour protection	
II	Monopile	Shallow (20-40m)	All	Driving pile<br>Scour protection	291
III	Tripot<br>Jacket	Median (5-50m), could be very deep	Soft clay with varying depth	Driving pile	763
IV	Semi-Submersible<br>Spar<br>TLP	Deep (>60m)	Not clear	Mooring installation	22

References

1. Negro, V.; López-Gutiérrez, J.S.; Esteban, M.D.; Alberdi, P.; Imaz, M.; Serraclara, J.M. Monopiles in offshore wind: Preliminary estimate of main dimensions. Ocean Engineering **2017**, 133, 253-261.

2. Li, C.; Mogollón, J.M.; Tukker, A.; Steubing, B. Environmental impacts of global offshore wind energy development until 2040. Environmental Science & Technology **2022**, 56(16), 11567-77.

3. Schielzeth H.; Dingemanse N.J.; Nakagawa S.; Westneat D.F.; Allegue H.; Teplitsky C.; Réale D.; Dochtermann N.A.; Garamszegi L.Z.; Araya-Ajoy Y.G. Robustness of linear mixed-effects models to violations of distributional assumptions. Methods in ecology and evolution **2020**, 11(9), 1141-52.

4. Zuur A.F.; Ieno E.N.; Elphick C.S. A protocol for data exploration to avoid common statistical problems. Methods in ecology and evolution **2010**, 1(1), 3-14.

5. Horwath E.S.; Hassrick J.; Grismala R.; Diller E. Comparison of Environmental Effects from Different Offshore Wind Turbine Foundations. Rep. ICF OCS Study BOEM. **2020**, 41-53.

6. Tsai L.; Kelly J.C.; Simon B.S.; Chalat R.M.; Keoleian G.A. Life Cycle Assessment of Offshore Wind Farm Siting: Effects of Locational Factors, Lake Depth, and Distance from Shore. Journal of Industrial Ecology **2016**, 20(6), 1370-83.
